# Association of Cardiotoxicity With Doxorubicin and Trastuzumab: A Double-Edged Sword in Chemotherapy

**DOI:** 10.7759/cureus.18194

**Published:** 2021-09-22

**Authors:** Mohanad Gabani, Diana Castañeda, Quynh My Nguyen, Soo-Kyoung Choi, Cheng Chen, Ayesha Mapara, Adam Kassan, Alexis A Gonzalez, Tahsin Khataei, Karima Ait-Aissa, Modar Kassan

**Affiliations:** 1 Internal Medicine, Harlem Hospital Center, New York, USA; 2 Basic Sciences, California State University, Los Angeles, USA; 3 Skaggs School of Pharmacy and Pharmaceutical Sciences, University of California, San Diego, San Diego, USA; 4 Physiology, Yonsei University, Seoul, KOR; 5 Department of Emergency and Critical Care, Shanghai General Hospital, Shanghai, CHN; 6 Biological Sciences, Northeastern Illinois University, Chicago, USA; 7 School of Pharmacy, West Coast University, Los Angeles, USA; 8 Instituto de Química, Pontificia Universidad Católica de Valparaíso, Valparaiso, CHL; 9 College of Medicine, University of Iowa, Iowa City, USA; 10 Physiology, The University of Tennessee Health Science Center, Memphis, USA

**Keywords:** cancer, chemotherapy, trastuzumab, doxorubicin, cardiotoxicity

## Abstract

Anticancer drugs play an important role in reducing mortality rates and increasing life expectancy in cancer patients. Treatments include monotherapy and/or a combination of radiation therapy, chemotherapy, hormone therapy, or immunotherapy. Despite great advances in drug development, some of these treatments have been shown to induce cardiotoxicity directly affecting heart function and structure, as well as accelerating the development of cardiovascular disease. Such side effects restrict treatment options and can negatively affect disease management. Consequently, when managing cancer patients, it is vital to understand the mechanisms causing cardiotoxicity to better monitor heart function, develop preventative measures against cardiotoxicity, and treat heart failure when it occurs in this patient population. This review discusses the role and mechanism of major chemotherapy agents with principal cardiovascular complications in cancer patients.

## Introduction and background

According to the American Cancer Society, cancer is one of the most prevalent healthcare challenges worldwide with 1.7 million cancer diagnoses in the United States in 2017 [1]. Although advancements in cancer therapy have significantly decreased patient mortality [2,3], unfortunately, some cancer treatments can damage the heart, a condition known as cardiotoxicity. High blood pressure, arrhythmias, and heart failure can be caused or exacerbated by chemotherapy and radiation therapy, as well as by newer forms of cancer treatment such as targeted therapies and immunotherapies [4]. As a result, to treat patients holistically and facilitate a better prognosis, cardiologists and oncologists must use an interdisciplinary approach: patients exposed to anticancer treatments require cardiovascular evaluation, risk analysis, prevention and mitigation of cardiac injury and cardiotoxicity, and their cardiac function must be monitored during and long after the therapy. Thus, cardio-oncology is an emerging discipline [5] and an essential part of a comprehensive approach to cancer treatment.

## Review

Definition of cardiotoxicity

**Figure 1:**
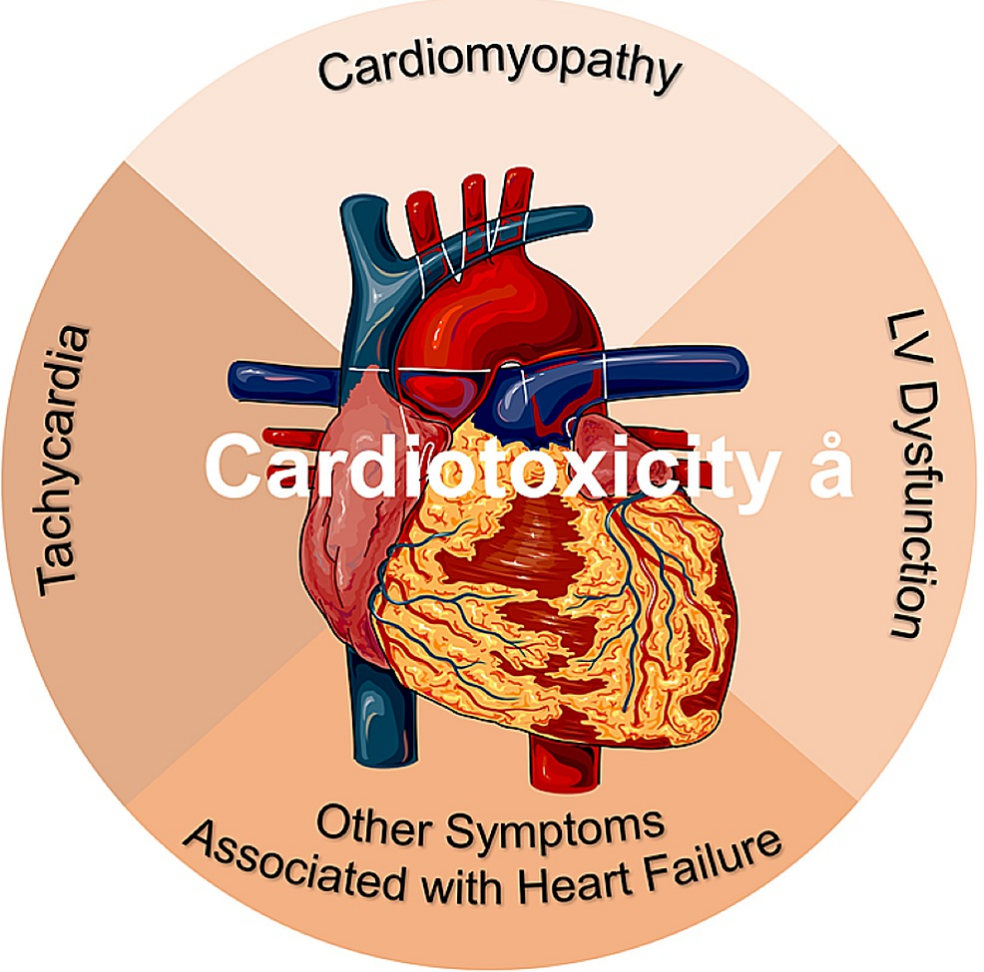
Cardiotoxicity. According to the Cardiac Review and Evaluation Committee, cardiotoxicity leads to cardiomyopathy, tachycardia, heart failure, and LV dysfunction. LV: left ventricular

Cardiotoxicity is a general term used to describe toxicity that can directly or indirectly affect the heart: directly, by damaging the heart structure; and indirectly, through thrombogenic states and hemodynamic alterations of blood flow [6]. The Cardiac Review and Evaluation Committee defines cardiotoxicity as the presence of one or more of the following conditions in patients who have received anticancer treatments [7]: (1) cardiomyopathy characterized by decreased left ventricular ejection fraction (LVEF) or the more severe abnormal ventricular septal motion; (2) heart failure symptoms; (3) tachycardia; (4) decrease in the minimum LVEF to less than 55% accompanied by signs and symptoms of heart failure (Figure 1) [7].

Additionally, according to the American Society of Echocardiography and the European Association of Cardiovascular Imaging [8], LVEF decrease can be categorized either as symptomatic or asymptomatic depending on its reversibility. Improvement to within 5% points of the baseline is considered reversible; improvement to ≥10% points from the nadir but remaining >5% points below the baseline is considered partially reversible; and improvement to <10% points from the nadir and remaining >5% points below the baseline is considered irreversible.

Furthermore, global systolic longitudinal myocardial strain (GLS) has been reported to accurately predict a subsequent decrease in LVEF [9]. A relative percentage GLS reduction of >15% from the baseline is considered abnormal and a marker of early left ventricular (LV) subclinical dysfunction. [10] However, neither GLS use nor its cutoff point to predict cardiotoxicity has been standardized [11].

Cardiotoxicity induced by chemotherapy

New therapies for managing neoplasms/tumors greatly extend the survival of cancer patients, in many cases making cancer a chronic pathology such as diabetes or systemic hypertension. Yet, these therapies have severe side effects [12].

**Figure 2:**
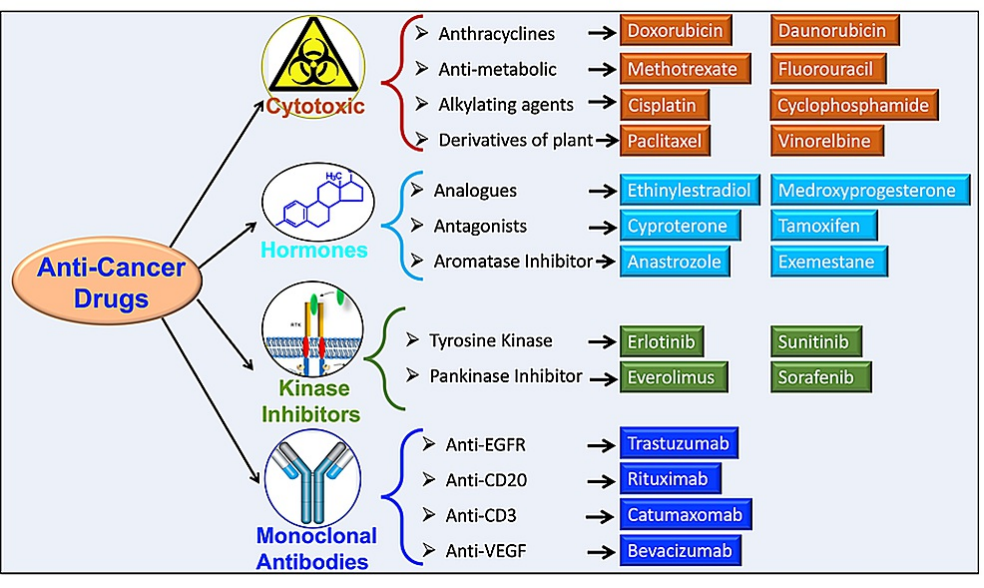
Classes of antineoplastic agents used as chemotherapy and representatives of each class. Anticancer drugs are divided into four groups: (1) cytotoxic (e.g., anthracyclines, antimetabolic, alkylating agents, and derivatives of plants); (2) kinase inhibitors (e.g., analogs, antagonists, and aromatase inhibitors; (3) hormones, (e.g., tyrosine kinase and pan-kinase inhibitors); and (4) monoclonal antibodies (e.g., trastuzumab, anti-EGF, anti-CD20, anti-CD3, and anti-VEGF). EGF: epidermal growth factor; CD: cluster of differentiation; VEGF: vascular endothelial growth factor

Chemotherapy is one of the most effective therapies for cancer treatment [2,3]. Figure 2 summarizes the classification of antineoplastic agents used as chemotherapy with representative examples of each class. Chemotherapy inhibits cell division through the action of many different types of cytotoxic drugs, hormonal agents, protein kinase inhibitors, and monoclonal antibodies. Yet, these agents are not only toxic to cancer cells they are also toxic to noncancerous tissues [13]. For the cardiovascular system, chemotherapy-induced complications were first reported in 1967 when pediatric leukemia patients, on high doses of anthracycline, developed heart failure [14].

Recently, it has been reported that doxorubicin, an anthracycline (Figure 2), can cause dose-dependent cardiotoxicity [15]. Along the same lines, a meta-analysis (based on several scientific journals, including the European Society of Cardiology, Association of Medical Scientific Societies of Germany, and the European Society of Medical Oncology) evaluated the cardiotoxicity of antineoplastic agents and how this limits their usefulness. This study reported that some cancer treatments are cardiotoxic and can trigger lethal complications as late as four years after treatment. The authors described that doxorubicin, given at a high dose (500 mg/m^2^), can cause cardiac complications in up to 36% of patients [16]. Similarly, the monoclonal antibody drug trastuzumab also causes cardiovascular toxic effects in up to 5% of patients. Therefore, clinicians need to be thoroughly aware of the cardiovascular toxic effects of anticancer drugs to be able to diagnose them early and not jeopardize the overall success of the treatment [7].

The individual management of patients requiring anthracyclines remains a challenge due to the uncertainty in cardiotoxicity predictors. A systematic review and meta-analysis of 18 studies regarding the incidence and chemotherapy predictors with anthracyclines in patients with cancer included 49,017 cancer patients, of whom 22,815 patients were treated with anthracyclines [17]. After an average follow-up of nine years, clinically evident cardiotoxicity occurred in 12% of patients, while subclinical cardiotoxicity developed in 24% of patients. Evaluation of the independent risk factors of cardiotoxicity showed that the cumulative doses of anthracycline were consistently an accurate and robust indicator of cardiotoxicity. Thus, anthracyclines present a significant risk of cardiotoxicity, especially when given at high cumulative doses [17].

To identify the effect of acute treatment with anthracycline on cardiotoxicity in children under 16 years of age with malignant childhood diseases, a cohort study was carried out among 110 children (between the ages of one month and 16 years) using anthracycline (doxorubicin). The incidence of anthracycline-induced cardiotoxicity was alarming. Within one month of doxorubicin treatment, the incidence of cardiac dysfunction was up to 14%, and after one year of treatment, the incidence increased to 25%. Thus, long-term follow-up is essential to diagnose late manifestations [18].

Another cohort study reviewed 105 breast cancer cases with anthracycline chemotherapy or a combination of anthracycline and the monoclonal antibody trastuzumab. One and four years after the start of chemotherapy, patients were clinically evaluated and tested with a baseline echocardiogram, as well as for systolic and diastolic function. Although subclinical, the incidence of myocardiopathy due to anthracycline was higher after four years following the first treatment (6%). Even more so, the combination of anthracyclines and trastuzumab further exacerbated the myocardial damage (more incidences of cardiomyopathy, diastolic dysfunction, and a greater drop in the LVEF) when compared to anthracyclines alone [19]. Diastolic dysfunction preceded or was associated with all cases of cardiomyopathy; therefore, more studies are required to determine whether diastolic dysfunction might be an early marker that identifies patients with a higher risk of developing cardiomyopathy.

These studies suggest that cardiotoxicity can occur within a wide window (as early as during treatment and as late as four years after the end of chemotherapy) [20], manifesting as many conditions: (1) acute or subacute, developing between the onset of treatment and two weeks after completion; and (2) chronic, developing at least one year after completion of therapy. Chronic cardiotoxicity can then be divided into two states: early chronic cardiotoxicity if it occurs during the first year after therapy and late cardiotoxicity if it occurs in the subsequent years after the end of therapy [21,22].

**Figure 3:**
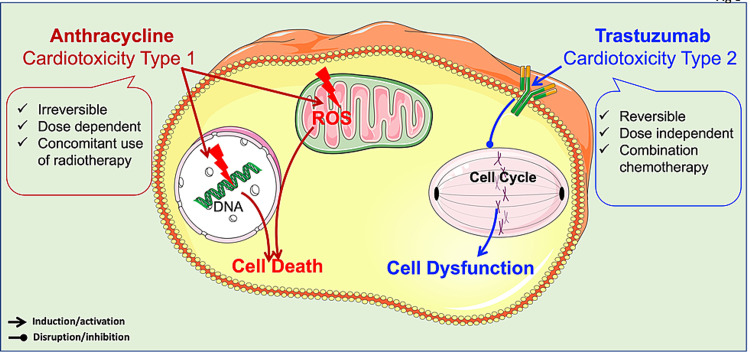
Types of cardiotoxicity. Cardiotoxicity is classified into type 1 and type 2. Type I is irreversible and dose-dependent, whereas type 2 is reversible and dose-independent.

Among the antineoplastic agents, the drugs most prone to cardiotoxicity are classified into two types (Figure 3): (1) Type 1: cardiotoxicity via anthracycline-like mechanisms. Its cardiac toxicity is dose-dependent and produces irreversible cardiac damage. (2) Type II: cardiotoxicity via trastuzumab-like mechanisms. These cause reversible cardiac damage, allowing chemotherapy to be halted until the patient recovers, and then restarted if indicated. This is achieved because there are no ultrastructural changes in myocytes [23,24].

In this review, we focus on the cardiotoxicity mechanisms produced by the treatment with anthracyclines or trastuzumab.

Anthracyclines and cardiotoxicity

Anthracyclines are a group of cytotoxic antibiotics that were initially extracted from the *Streptomyces* bacterium [25]. They are very effective drugs against a wide spectrum of solid and hematological malignancies and are a part of many treatment regimens [26]. Nevertheless, cardiotoxicity remains one of the main elements that limits their use. The main risk factors for developing cardiac failure from exposure to these cytotoxic agents are accumulated doses, age greater than 70, early or simultaneous irradiation, use of other drugs that damage the myocyte, and a history of heart diseases [27].

The mechanisms by which anthracyclines damage the heart are probably multifactorial [28]. The release of free radicals, alteration in iron homeostasis, changes in intracellular calcium, and mitochondrial dysfunction are some of the effects produced by anthracyclines [29]. The best-known mechanism is the pathway of damage mediated by free radicals. The reduction of the quinone group of the anthracycline generates a semiquinone radical that oxidizes rapidly, generating superoxide radicals that produce hydrogen peroxide. In turn, hydrogen peroxide interacts with the myocardium. Because the myocardium expresses a relatively lower amount of superoxide dismutase and catalase, its only defense is glutathione peroxidase, which itself is reduced by anthracyclines [30,31].

When ferric iron forms a complex with doxorubicin, it generates more free radicals, which, in turn, convert ferrous iron to ferric iron, a vicious circle that can damages mitochondrial and nuclear membranes, the cell membrane, and the endoplasmic reticulum, leading to an intracellular calcium decrease and reducing heart contractility [30,31].

Proinflammatory cytokines are also related to the cardiovascular side effects of anthracyclines because they induce the release of histamine, tumor necrosis factor-alpha, and interleukin-2, proteins that induce dilated cardiomyopathy in addition to beta-adrenergic dysfunction [32].

Although the previously mentioned mechanisms are widely studied, the interaction of reactive oxygen species (ROS) with cellular elements and the formation of free radicals (induced by anthracyclines) are not the mechanisms directly responsible for the cellular injury. This was demonstrated for the first time by Lyu et al. [33] who used mouse embryos exposed to anthracyclines and observed DNA breaks and cell death dependent on the presence of DNA topoisomerase II beta (TOP2B). Based on this finding, Zhang et al. conducted studies on cardiac tissue from wild-type (WT) versus TOP2B-knockout (KO) mice treated with doxorubicin. They demonstrated that the first step toward cardiomyocyte damage is independent of ROS and depends on a complex formed by the TOP2B-ROS (generated by anthracycline)-DNA. This complex leads to the suppression of transcription factors (via the activation of p53), DNA degradation, and inevitable apoptosis due to mitochondrial dysfunction [15]. In contrast to controls, the mutant animals (lacking TOP2B) exposed to doxorubicin did not display acute, chronic cardiac injury or the reduction of the LVEF [34]. Thus, the depletion of cardiac TOP2B should prevent doxorubicin-induced cardiotoxicity while preserving its tumor-killing effect.

Recently, several mechanisms have been described by which anthracyclines cause cardiotoxicity; these mechanisms are still under investigation. Anthracyclines show an affinity for cardiolipin, a cofactor of the respiratory chain enzymes (i.e., cytochrome c oxidase, NADH, and oxidoreductase). Cardiolipin possesses a high density of phospholipids and, thus, a higher affinity to anthracyclines, especially doxorubicin. A cardiolipin-doxorubicin complex can damage the inner mitochondrial membrane and inhibit oxidative phosphorylation, thereby losing its function as a cofactor [35]. Another cell component with which anthracyclines interact to cause cardiotoxicity is the protein titin. This protein comprises part of the sarcomere in striated muscle, serving as a scaffold for the assembly of myofilament proteins in the sarcomere, as well as mediating the passive contractile forces [36]. Anthracyclines degrade titin and alter sarcomeric cardiac structure (sarcopenia) through the loss and disorganization of sarcomere myofibrils, sarcoplasmic reticulum dilatation, mitochondrial edema, and cytoplasmic vacuolization. Therefore, titin degradation can lead to progressive diastolic and systolic dysfunction, with the suppression of transcription of sarcomere proteins [37,38]. In addition, anthracyclines deplete reserves of GATA binding protein 4 (GATA4), a transcriptional factor that regulates the apoptotic pathway and preserves mitochondrial function, and is a potent regulator of cardiac gene activity [39].

**Figure 4:**
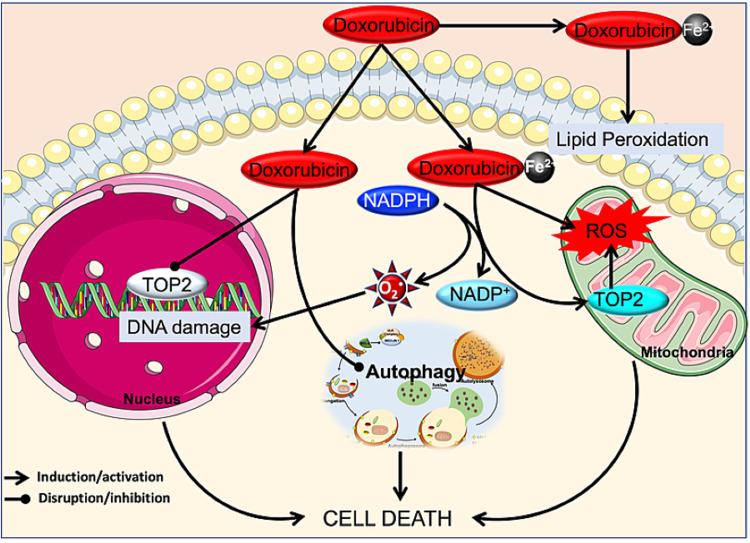
Cardiotoxicity by doxorubicin. Doxorubicin acts on TOP2B causing breaks in genomic and mitochondrial DNA, and, as a consequence, elevating ROS. Additionally, ferric iron forms a complex with doxorubicin that generates even more free radicals via NADPH oxidase and lipid peroxidation. Furthermore, doxorubicin disrupts autophagy flux in cardiomyocytes by impairing lysosome acidity and function. All these mechanisms impair cardiomyocytes and are cardiotoxic. TOP2B: DNA topoisomerase II beta; ROS: reactive oxygen species. O_2_^.^: superoxide anion. Fe^2+^: ferric anion. NADPH: nicotinamide adenine dinucleotide phosphate

Furthermore, doxorubicin can contribute to cardiotoxicity by disrupting autophagy, a programmed cell death pathway, independent of apoptosis and necrosis. In a doxorubicin-induced heart failure model in rats, it has been concluded that doxorubicin damages the mitochondria of cardiomyocytes, which then leads to heart failure through the induction of pathological autophagy. The inhibition of autophagy has reduced doxorubicin-induced mitochondrial injury and rescued heart function [40]. Similarly, in vitro treatment of cardiomyocytes with doxorubicin induces autophagy and leads to cardiomyocyte death [41]. Overexpressing GATA4 in the cardiomyocytes inhibited doxorubicin-induced autophagy, reducing cardiomyocyte apoptosis [42]. In contrast, it has been shown that activation of autophagy through starvation prior to doxorubicin administration mitigates the acute cardiotoxicity of this drug. This mitigation may occur partly because fasting restores and strengthens myocardial autophagic flux, which reduces the negative impact of doxorubicin on the cardiomyocytes. Based on these findings, patients on doxorubicin might be able to prevent or reduce the risk of cardiotoxicity through fasting or caloric restriction [43,44]. Additionally, induction of autophagy using rapamycin has shown a prospective cardioprotective role against doxorubicin-induced cardiotoxicity. This highlights rapamycin as a plausible adjuvant therapy possessing a high therapeutic index to counteract and improve the life-threatening impediment of doxorubicin actions in clinical practice [45]. The discrepancy between these studies might be due to the use of doxorubicin either in vivo or in vitro. Furthermore, the majority of the studies in the literature use short-term and high-dose doxorubicin exposure, which does not reflect the chronic clinical use of doxorubicin. Li et al. overcame this issue by giving multiple injections of doxorubicin at doses used clinically [46] to demonstrate that treatment with clinical doses of doxorubicin blocks autophagy flux in cardiomyocytes by impairing lysosomal acidity and function. Thus, the reduction of autophagy induction protects against doxorubicin-induced cardiotoxicity. Even though numerous studies have investigated molecular mechanisms of doxorubicin cardiomyopathy, a single, unifying model of pathogenesis remains elusive. Thus, there is a need to establish a specific model that mimics the clinical dose of doxorubicin in vivo using different animal models. The mechanism of action of doxorubicin is summarized in Figure 4.

Trastuzumab and cardiotoxicity

Although the pathophysiology of cardiac dysfunction associated with trastuzumab is not entirely clear, various mechanisms have been proposed to explain it. On one hand, trastuzumab, as a monoclonal antibody, can initiate antibody-dependent cellular cytotoxicity that can affect cardiomyocytes, increasing cardiac toxicity [47,48]. On the other hand, various arguments support the idea that human epidermal growth factor 2 (HER2) contributes to cardiotoxicity [49]. Trastuzumab inhibits the proliferation of human tumor cells overexpressing the HER2 receptor that is expressed on cardiac cells and plays an essential role in the proliferation, growth, and survival of cardiomyocytes; thus, the HER2 signaling pathway controls cardiac development and function [50] and is essential to prevent the development of cardiomyopathy. The deletion of HER2 has led to multiple features of dilated cardiomyopathy, including cavities dilation, thinning of the cardiac wall, and decreased contractility [50]. In the absence of HER2 function, cardiomyocytes are not able to activate survival pathways and thus accumulate ROS, leading to cardiac dysfunction [51]. Additionally, reduction of HER2 activity dampens the extracellular signal-regulated kinase (MEK/ERK) signaling pathway, inducing apoptosis [52]. MEK/ERK inhibition through the trastuzumab-induced reduction in HER2 leads to an increase in the number of mitochondrial permeability transition pores (mPTP) with subsequently increased sensitivity to Ca^2+^ overload, excessive production of cytotoxic ROS, and suppressed gap junction permeability. These factors culminate in myocyte injury [53]. Additionally, patients with a mutation that regulates the MEK/ERK activity display hypertrophic cardiomyopathy [54]. Overall, these findings indicate that trastuzumab regulates MEK/ERK pathway, a pathway important to stimulate proliferation and survival and to protect the function of myocytes [55].

The ratio between antiapoptotic and proapoptotic stimuli is a key regulator of mitochondrial function in cardiomyocytes. Trastuzumab induces cardiotoxicity by downregulating the antiapoptotic protein, B-cell lymphoma-extra large (BCL-XL), and by upregulating the proapoptotic protein B-cell lymphoma-extra small (BCL-XS). [56]

Phosphoinositide 3-kinase (PI3K) and mammalian target of rapamycin (mTOR) are key elements in HER2 downstream signaling. The PI3Ks phosphorylate phosphatidylinositol 4,5 bisphosphate (PIP2) to phosphatidylinositol 3,4,4-triphosphate (PIP3), which, in turn, leads to the phosphorylation of Akt, a serine/threonine kinase which has an impact on cancer cell cycling, survivalc, and growth [57-59]. mTOR is a serine/threonine protein kinase, which is found downstream of PI3K. The role of the PI3K/mTOR in cardiac structure and function is very well established [60]; therefore, the deregulation of this pathway by trastuzumab leads to cardiotoxicity.

During cancer, when HER2 is overexpressed, mitogen-activated protein kinase (MAPK) becomes hyperactive [61]. Abnormalities in the MAPK signaling pathway play a critical role in the development of cancer [62]. However, MAPK signaling also drives the pathogenesis of cardiac diseases such as cardiac hypertrophy and cardiac remodeling after myocardial infarction [63]. Because of this important role, the cardiotoxicity of trastuzumab may be related to the drug’s inhibition of MAPK.

**Figure 5:**
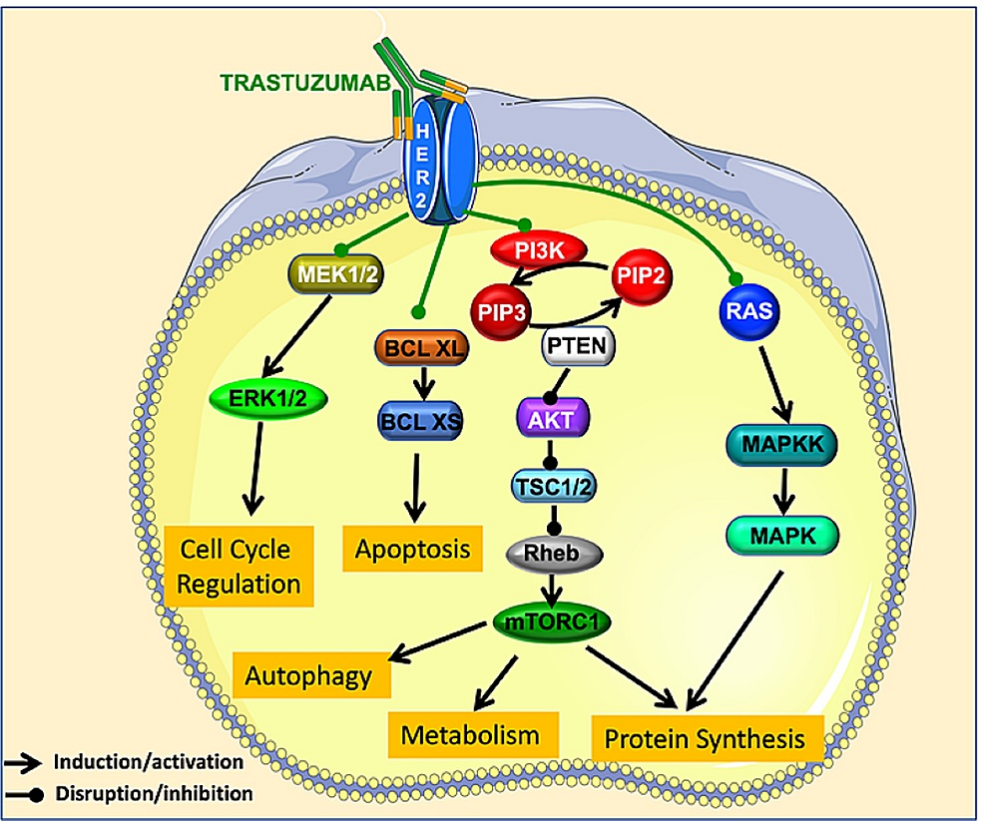
Cardiotoxicity by trastuzumab. Trastuzumab acts on HER2, decreasing signaling via the MEK/ERK pathway and causing apoptosis. It also downregulates the antiapoptotic protein BCL-XL and upregulates the proapoptotic protein BCL-XS; impairs the PI3K/mTOR pathway, causing decrease in autophagy and protein synthesis; and decreases the MAPK activity, dampening protein synthesis. Together, these impairments are cardiotoxic HER2: human epidermal growth factor 2; BCL-XL: B-cell lymphoma-extra large; BCL-XS: B-cell lymphoma-extra small; PIP2: phosphorylate phosphatidylinositol 4,5 bisphosphate; PIP3: phosphatidylinositol 3,4,4-triphosphate; MAPK: mitogen-activated protein kinase; mTOR: mammalian target of rapamycin

Clinically, the cardiotoxic effects of trastuzumab can manifest as asymptomatic (decreases in LVEF) or symptomatic (congestive heart failure) which can lead to death [64]. The mechanism of action of trastuzumab is summarized in Figure 5.

Prevention and cardiotoxicity treatment

To date, as there are no specific treatments for cardiac insufficiency induced by chemotherapy, the treatment of choice for congestive heart failure remains angiotensin-converting enzyme inhibitors ACEI (enalapril), beta-blockers (metoprolol), and diuretics (carvedilol). For instance, enalapril and metoprolol have been shown to reduce cardiotoxicity in patients with elevated concentrations of troponin I due to chemotherapy. These agents prevented the reduction in the LVEF [65]. Numerous basic science studies and clinical trials have shown that administration of ACEI had a cardioprotective effect with reduced morbidity and mortality in animal models and patients of acute and chronic chemotherapy-induced cardiotoxicity [66,67]. However, the sample size used in all studies that aimed to evaluate the preventive effect of ACEI on the heart during chemotherapy is small and needs to be increased to confirm the preventive outcome. Additionally, it has been suggested that beta-blockers can prevent trastuzumab-related cardiotoxicity by promoting ERK signaling [68]. However, more studies are needed to further evaluate the cardioprotective effects of beta-blockers in chemotherapy-induced cardiotoxicity.

Although some of these drugs attenuate the decrease in LVEF, they have no effect on GLS or cardiac biomarkers. Even though GLS may be a more sensitive tool to detect early cardiotoxicity because there is no specific standardized guidance for GLS [11], it is recommended that cancer treatment should not be stopped, interrupted, or reduced in dose based on GLS reduction alone [10]. Recently, other treatments have been introduced such as antioxidant drugs (probucol), bioactive compounds (inorganic nitrates), and aerobic exercise training.

Anthracycline-induced cytotoxicity is mainly due to the generation of ROS in the cardiomyocytes which results in LV dysfunction. In animal models of cardiotoxicity associated with doxorubicin, the antioxidant probucol was effective in preventing LV dysfunction and could be a potential therapeutic tool to combat cardiotoxicity [69]. Additionally, carvedilol is prescribed as cardioprotective due to its antioxidant properties via increasing levels of GATA4 [70]. Along the same lines, ranolazine, a drug used to treat chronic angina, showed cardioprotective effects in both animals and patients undergoing chemotherapy by suppressing ROS production [71-73]. Recently, cardio-oncology research has been focusing on the redox compound hydrogen sulfide (H_2_S). Exogenous H_2_S has been shown to protect against cardiotoxicity; however, less is known about its adverse effect so further basic and clinical research will be needed to assess the safety of H_2_S [74,75]. Nevertheless, not all antioxidants are equally effective. Vitamin E, also known for its antioxidant effects, appears not to prevent ventricular dysfunction in long-term experimental and clinical trials [71].

Dexrazoxane is an iron chelator known to prevent the formation of excess hydroxyl radicals, thereby decreasing the incidence of cardiac insufficiency and boosting LV function. Dexrazoxane is the only Food and Drug Administration-approved drug for chemotherapy-induced cardiotoxicity. Its protective effect was observed in patients receiving high doses of doxorubicin (300 mg/m_2_). One of its protective mechanisms is associated with suppression of anthracycline-induced troponin elevation, a key player in myocytes death. Another mechanism by which dexrazoxane likely produces its beneficial effect is through preventing doxorubicin binding to TOP2B, thereby preventing DNA breaks and cell death [39,76]. It has been reported that dexrazoxane may attenuate the chemotherapeutic efficacy of doxorubicin but recent studies have shown that dexrazoxane does not interfere with the antitumor activity nor does it reduce the progression or overall survival, which are the key endpoints of cancer studies [75].

Other reports showed that there is an association between dexrazoxane and induction of a second tumor [76]. These results were questionable, especially because the statistical method applied in these studies was not appropriate and importantly because at a clinical level the examination and the follow-up of more than 1,000 patients showed that this association was not confirmed [77,78]. Based on the clinical data outcome, dexrazoxane could present as the most appropriate cardioprotective drug to use in combination with anticancer drugs.

Inorganic nitrate is a bioactive compound that can be reduced into nitrite and nitric oxide in vivo. Once reduced, it could have therapeutic properties for diseases related to nitric oxide bioavailability disruption. Recently, it was shown that administration of inorganic nitrates to mice during doxorubicin therapy (at a rate of 400% of what is recommended by the World Health Organization) decreased ventricular dysfunction, cell death, oxidative stress, and mitochondrial damage, without reducing the antineoplastic effect of doxorubicin [79].

Another way to reduce anthracycline and trastuzumab cardiotoxicity is with aerobic exercise. In addition to its many benefits in cancer management [80-82], aerobic exercise training has been shown to increase systolic and diastolic function, diminishing pathological restructuring of cardiac tissue, thereby preventing dilatation of the left ventricle. At the same time, aerobic exercise increases resistance to fatigue in patients with cardiac insufficiency [83-85]. It also protects the heart against oxygen free radicals by activating endogenous antioxidant processes and increasing the expression of antioxidant enzymes that decrease their production [69]. Dolinsky et al. have demonstrated that intense aerobic exercise training for only eight weeks decreased doxorubicin-induced cardiac damage [86]. Aerobic exercise training has not only attenuated the adverse LV remodeling but also reduced the level of atrial natriuretic peptide and lipid peroxidation byproducts [85]. Additionally, aerobic exercise can regulate proapoptotic signals by decreasing the expression of p53 (an apoptotic mediator) and increasing GATA4 [70]. Although several studies have obtained positive results after testing the role of aerobic exercise in preventing chemotherapy-induced cardiotoxicity [86,87], we need more information to define in greater detail the effects of aerobic exercise as a means of preventing cancer therapy-induced cardiotoxicity [88].

Another way to prevent and monitor the early signs of cardiotoxicity is echocardiographic measurements. These advanced echocardiographic measurements are preferred, when available, to serve as the basis for clinical decisions when performed with adequate expertise performing cardiac safety studies [89]. In the clinical setting, cardiac imaging surveillance is used for the early detection of cardiotoxicity. Recent advances in molecular imaging of apoptosis and tissue characterization by cardiac magnetic resonance imaging (MRI) allow early detection of patients at high risk for developing cardiotoxicity prior to a drop in LVEF. Therefore, cardiac MRI is the gold standard for determining cardiac volumes and function because of the superior image quality [90]. Current guidelines recommend this imaging modality for the confirmation of cancer therapy-related cardiac dysfunction, mainly when echocardiographic- or radionuclide-derived LVEF is uncertain [8].

**Figure 6:**
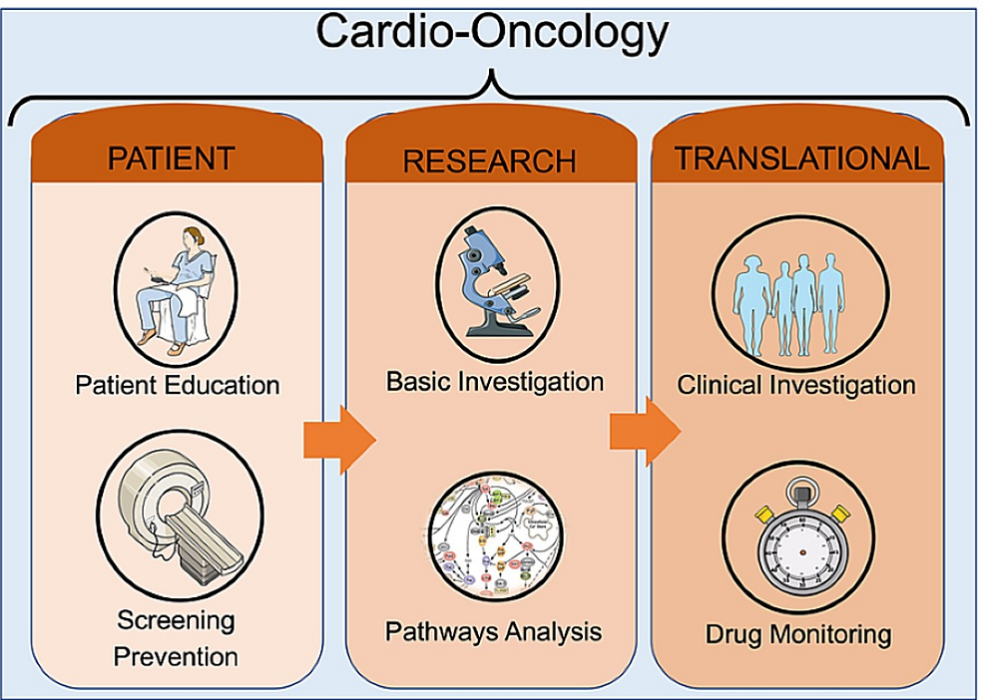
A comprehensive plan for the cardio-oncologist to address comorbidities when personalizing treatment for a cancer patient. Patient education and drug screening are key because they allow the cardio-oncologist to detect cardiotoxicity in its earliest stages. Additionally, pathway analysis should point to new techniques for managing cardiovascular disease in cancer patients. Finally, translational investigation and drug monitoring must be incorporated into any cardio-oncology program.

With the increasing number of cancer survivors, often burdened with pre-existing or new cardiovascular disease or risk factors, the need has arisen for a new specialty in the field of cardiovascular care that can assess and treat these patients. This specialty must combine cardiologists and oncologists. In the same way, all healthcare providers involved in the care of patients with cancer and heart disease should be fully aware of the adverse impact of cardiovascular disease on the survival of these patients. Collaboration is necessary to mitigate the effect of cardiovascular toxicity associated with these anticancer therapies that otherwise save lives. Cardio-oncologists can play a fundamental role in combining the two specialties by creating a comprehensive plan to address comorbidities and providing guidance for choosing the optimal treatment. The cardio-oncologist unit should focus on three aspects: (a) patient education and drug screening, which is a way to detect the cardiotoxicity in its earliest stages; (b) basic investigation and pathway analysis that addresses cardiotoxicity as a consequence of cancer therapy and discusses the prevention, diagnosis, and management of cardiovascular disease in patients with cancer; and (c) translational clinical investigation and drug monitoring. These aspects and their importance to the cardio-oncology field are summarized in Figure 6.

## Conclusions

Cardiotoxicity represents a side effect for patients receiving chemotherapy and increases with other concomitant risk factors; however, in some cases, it can be prevented or limited. The methods to combat cardiotoxicity range from pharmacological interventions that limit cardiac restructuring to nonpharmacological ones such as aerobic exercise, which gives us a simple method of preserving the cardiac function in exposed patients. New methods are needed to provide novel and promising instruments for the detection of cardioprotective gene modulators that are affected by antineoplastic drugs. This could lead to a change in the current definition of cardiotoxicity from a clinical definition to a subclinical one based on earlier, sensitive, and specific biomarkers.
